# Prognosis of Sentinel Node Staged Patients with Primary Cutaneous Melanoma

**DOI:** 10.1371/journal.pone.0029791

**Published:** 2012-01-19

**Authors:** Otmar Elsaeßer, Ulrike Leiter, Petra G. Buettner, Thomas K. Eigentler, Friedegund Meier, Benjamin Weide, Gisela Metzler, Helmut Breuninger, Claus Garbe

**Affiliations:** 1 Center for Dermatooncology, Department of Dermatology, and Central Malignant Melanoma Registry of the German Dermatological Society, Eberhard-Karls-University of Tuebingen, Tuebingen, Germany; 2 Skin Cancer Research Group, School of Public Health and Tropical Medicine, James Cook University, Townsville, Queensland, Australia; The University of Queensland, Australia

## Abstract

**Background:**

This study investigated survival probabilities and prognostic factors in sentinel lymph node biopsy (SLNB) staged patients with cutaneous melanoma (CM) with the aim of defining subgroups of patients who are at higher risk for recurrences and who should be considered for adjuvant clinical trials.

**Methods:**

Patients with primary CM who underwent SLNB in the Department of Dermatology, University of Tuebingen, Germany, between 1996 and 2009 were included into this study. Survival probabilities and prognostic factors were evaluated by Kaplan-Meier and multivariate Cox proportional hazard models.

**Results:**

1909 SLNB staged patients were evaluated. Median follow-up time was 44 months. Median tumor thickness was 1.8 mm, ulceration was present in 31.8% of cases. The 5-year Overall Survival (OS) was 90.3% in SLNB negative patients (IB 96.2%, IIA 87.0%, IIB 78.1%, IIC 72.6%). Patients with micrometastases (stage IIIA/B) had a 5-year OS rate of 70.9% which was clearly less favorable than for stages I–II. Multivariate analysis revealed tumor thickness, ulceration, body site, histopathologic subtype and SLNB status as independent significant prognostic factors.

**Conclusion:**

Survival rates of patients with primary CM in stages I–II were shown to be much more favorable than previously reported from non sentinel node staged collectives. For future clinical trials, sample size calculations should be adapted using survival probabilities based on sentinel node staging.

## Introduction

Sentinel lymph node biopsy (SLNB) is a minimally invasive procedure with minor morbidity for patients with cutaneous melanoma (CM). SLNB allows to ascertain the status of the regional node field and assists with exact staging [1]–[3]. During the last decade SLNB has become a routinely performed procedure in most melanoma centers worldwide [4]–[6] There is international consent that SLNB should be discussed with and recommended to patients when at least one of the following indications is present [1]: (1) the risk of clinically occult nodal metastases is sufficient to justify the procedure (approximately 10%); (2) the prognostic information from SLNB would be of value to the patient and the treating physicians; (3), the tumor status of the SLN would be useful in guiding decisions regarding complete lymphadenectomy and adjuvant therapy; (4) nodal staging information is important for entry into clinical trials if the patient is interested; and/or (5) the risks of SLNB are acceptable to the physician and the patient [7]. SLNB is regarded to be a valuable procedure for CM patients allowing to stage regional lymph nodes with little morbidity [5], [8].

The accuracy of SLNB staging has been shown through a long-term follow-up of SLNB negative patients. These patients have an improved survival compared to the SLNB positive group, and have less regional recurrences in the mapped node fields [3]. In SLNB staged patients nodal recurrences seem to occur less frequent but so far recurrence rates were reported in only a few case series, some of which had limited follow-up [4], [9]–[14]. As the presence of nodal micrometastases is the single most important prognostic factor [5] patients want to get this information to be considered for new therapies under evaluation in clinical trials, and to make an informed decision about complete lymphadenectomy and adjuvant therapy. In addition, the information provided by a positive SN can be used to counsel patients regarding enrollment into clinical trials and can serve as the basis for discussing screening and follow-up regimen [15].

So far, few data on survival probabilities and prognostic factors in SLNB staged patients were reported. In order to validate the AJCC classification, Balch and Co-workers evaluated stage I/III CM patients who were considered as clinically node negative [16]. SLNB staging had been performed for a part of these patients and there are only few cohorts of melanoma patients with a long-term follow-up after negative SLNB staging. The present study was performed to evaluate survival probabilities and prognostic factors of 1909 SLNB staged CM patients with the aim to define groups of patients who are at higher risk for recurrences and who should be considered for adjuvant clinical trials and undergo a closer follow-up.

## Methods

The present analysis included patients with cutaneous melanoma (CM) diagnosed and treated by the Department of Dermatology, University Tuebingen, Germany. Patients included were diagnosed with incident invasive (Clark's level of invasion II or more) primary CM between January 1^st^ 1996 and June 30^th^ 2009. All patients had given their written informed consent (Supporting Information S1), the local Ethic committee statement had no concern (Supporting Information S2). At the University Department of Dermatology in Tuebingen, Germany, sentinel lymph node biopsy was introduced in January 1996 and has been routinely performed by four dermato-surgeons over the entire time period in all CM patients with a tumor thickness of 1.00 mm or more. SLNB was also performed in 101 patients with smaller tumors if additional unfavorable prognostic factors as a level of invasion IV–V, ulceration or tumor regression were present.

Follow-up examinations were performed according to the recommendations of the German Society of Dermatology comprising physical examinations every three months during years 1–5 after primary tumor diagnosis, twice yearly in years 6–10 and, twice respectively once yearly, lymph node ultrasound and blood tests [17]. Body site of the primary melanoma was classified into five anatomical sites: head with scalp and neck, anterior trunk, posterior trunk, upper and lower extremities. Histopathological analysis of sentinel lymph nodes was based on four serial sections performed at each of two levels. The sections from each level were stained with H&E and immunohistochemical stains for S-100 protein, HMB-45, and Melan-A. Reports of the responsible dermato-histopathologists were documented. SLNB containing isolated positive tumor cells or micrometastases of ≤0.1 mm were not judged as positive, in agreement with the 2002 AJCC melanoma classification [18].

Recurrences during follow-up were distinguished as loco-regional metastases (satellite/in-transit metastases), regional lymph node metastases and distant metastases. Loco-regional, nodal and distant recurrences were analyzed. Satellite metastasis was defined as recurrence in the first melanoma field 2 cm of the edge of the wide excision margin. In-transit metastasis was defined as subcutaneous metastases from 2 cm to the first nodal site. Only the first recurrence was considered for this analysis. False-negative SNBs were defined as procedures in which the initial histopathologic evaluation was negative, but the patients tumor recurred in the same node field. Patients whose tumor recurred as satellite or in-transit disease, followed by regional node field recurrence, were not considered to have a false-negative SNB on the assumption that the disease may not have been present in the regional nodes at the time of biopsy.

### Statistical analysis

Statistical analyses were performed with the statistic software SPSS 19 (PASW, IBM SPSS, Chicago, IL, USA). Numerical variables were described by mean value and standard deviation (SD) if approximately normally distributed or median value and inter-quartile range (IQR) if skewed. Proportions were presented with 95% confidence intervals (95%-CI).

The time between primary excision of histological diagnosed CM and the date of the last follow-up visit or the date of death was used to calculate the follow-up time for melanoma-specific overall survival (OS), and the date of first recurrence for the disease-free survival (DFS), respectively. Only deaths due to CM (melanoma specific deaths) were considered “events”. In case that mortality and cause of death was not directly reported, registration offices were systematically addressed. Survival probabilities with 95%-CI were calculated according to Kaplan-Meier and compared with log-rank test statistics. Multivariate Cox proportional hazard models were calculated to judge significant independent prognostic factors. Forward and backward stepwise procedures of the multivariate modeling process resulted in the same model. Results of Cox proportional hazard modeling were described as relative risks (hazard ratios) together with 95%-CI and p-values. Throughout the analysis, p-values less than 0.05 were considered as statistically significant.

## Results

### Selection of patients

From January 1996 to June 2009, a total of 6,028 patients with primary cutaneous melanoma were documented by the Department of Dermatology, Tuebingen, Germany. Patients with advanced disease, unknown primary or melanoma in extra-cutaneous localization (n = 660), patients without SLNB (n = 3,330) and patients with follow-up less than 3 months (n = 129) were excluded. The present cohort consisted of 1,909 SLNB staged patients, of these 1,697 (88.9%) with negative and 212 (11.1%) with positive SLNB. Of 1,697 SLNB negative patients, 99 (5.8%) presented with a tumor thickness of less than 1.00 mm while there were two (0.9%) of 212 SLNB positive patients with a tumor thickness below 1 mm. Before SLNB was performed all patients underwent physical examinations and lymph node ultrasound. If these examinations suggested metastases these findings were judged as macrometastases as they were detected by clinical methods, and these patients were not included in the present analysis.

### Description of sample

The collective of 1909 SLNB staged CM patients consisted of 53.0% males and 47.0% females. The mean age at diagnosis was 55.9 years (SD±16.1), the median tumor thickness was 1.80 mm (IQR = [1.2, 2.8]). Ulceration was present in 31.8% of the primary lesions (Table 1). The total rate of patients with recurrences was 20.2% (N = 385), 35.1% of the first recurrences were satellite/intransit metastases (N = 135), 30.9% were regional lymph node metastases (N = 119) and 34.0% were distant metastases (N = 131). If metastases on different sites occurred simultaneously, the metastasis with the worst prognosis counted as first metastasis. The rate of false negative SLN for the entire series was 13.1%. Disease related deaths occurred in 10% (n = 190; 95%CI = 8.7, 11.4). Follow up information for a period of 5-year or more was available for 35.3% of patients, 52% of all patients were diagnosed later than 2004 and did not yet reach a five-years follow-up at the timepoint of the analysis. The median OS time for the whole collective was 44 months (IQR = 21, 74); while the median DFS was 38 months (IQR = 15, 68).

**Table 1:** Five-year **overall survival** (OS) and **disease-free** survival (DFS) probabilities based on Kaplan-Meier in sentinel node staged patients with CM (n = 1909).

Prognostic Factor	Number of Patients n (%)	Censored (%) at OS	P value 5-year OS [95% CI]	Censored (%) at DFS	P-value 5-year DFS [95% CI]
**Thickness**			P<0.0001		P<0.001
≤1.00 mm	188 (9.8%)	96.8	97.5 [94.6, 100]	94.1	96.9 [94.2, 99.6]
1.01–2.00 mm	934 (48.9%)	93.9	93.0 [90.7, 95.3]	86.6	84.2 [81.3, ,87.1]
2.01–4.00 mm	565 (29.6%)	86.2	82.8 [78.7, 86.9]	71.9	64.2 [59.7, 68.7]
>4 mm	222 (11.6%)	77.9	68.3 [60.1, 76.5]	61.7	55.3 [47.1, 63.5]
**Ulceration**			P<0.0001		P<0.001
absent	1094 (68.2%)	93.3	93.2 [91.3, 95.1]	87.3	86.2 [83.9, 88.5]
present	511 (31.8%)	83.0	75.8 [70.9, 80.7]	66.9	57.7 [52.5, 62.9]
**Body site**			P<0.0001		P<0.001
Head and Neck	231 (12.1%)	91.3	88.7 [83.0, 94.4]	79.1	75.5 [68.5, 82.6]
Trunk	729 (38.2%)	87.0	84.3 [81.0, 87.6]	78.1	74.1 [66.3, 78.0]
Upper limb	343 (18.0%)	97.1	95.6 [92.7, 98.5]	91.3	89.2 [85.1, 93.3]
Lower limb	606 (31.7%)	89.3	88.7 [85.6, 91.8]	76.4	72.6 [68.5, 76.7]
**AJCC Stage**			p<0.001		p<0.001
I A	90 (5.3%)	100.0	100.0	95.2	97.2 [95.2, 99.2]
I B	824 (43.2%)	96.0	96.2 [94.4, 98.0]	90.9	90.3 [89.0, 91.6]
II A	434 (22.7%)	90.6	87.0 [82.9, 91.1]	80.6	75.4 [72.9, 77.9]
II B	260 (13.6%)	83.8	78.1 [71.2, 85.0]	67.7	58.2 [54.2, 62.2]
II C	87 (4.6%)	80.5	72.6 [59.5, 83.9]	59.8	50.7 [43.0, 58.4]
III A	119 (6.2%)	73.1	72.6 [62.8, 82.4]	53.8	45.9 [40.4, 51.4]
III B	95 (5.0%)	73.7	65.6 [53.4, 77.8]	54.7	44.9 [38.7, 51.1]
**Age**			P = 0.035		P = 0.001
< = 45-year	531 (27.8%)	92.1	92.0 [89.3, 94.7]	84.0	82.6 [78.9, 86.3]
46–60 years	529 (27.7%)	87.7	86.3 [82.8, 89.8]	78.8	76.8 [72.7, 80.9]
61–70 years	454 (23.8%)	88.1	85.0 [80.7, 89.3]	78.0	73.7 [68.8, 78.6]
>70 years	395 (20.7%)	92.7	88.0 [83.1, 92.9]	79.0	70.2 [64.1, 76.3]
**Gender**			P = 0.068		P = 0.135
Male	1012 (53.0%)	88.8	86.2 [79.6, 93.0]	78.8	74.6 [74.5, 77.9]
Female	897(47.0%)	91.4	89.5 [87.0, 92.0]	81.6	78.0 [74.7, 81.3]
**Clark-Level**			P<0.0001		P<0.001
Level II	12 (0.7%)	100.0	100.0	91.7	85.7 [80.7, 88.2]
Level III	209 (12.6%)	95.7	93.1 [91.0, 95.1]	89.0	88.2 [83.3, 93.1]
Level IV	1350 (81.4%)	90.8	89.1 [88.0, 90.2]	80.6	76.9 [76.8, 79.6]
Level V	88 (5.3%)	75.0	82.3 [79.1, 85.5]	60.2	55.1 [43.0, 67.2]
**Histological Subtype**			P<0.001		P<0.001
SSM	993 (54.6%)	90.2	91.0 [89.9, 92.1]	82.6	80.6 [79.5, 81.7]
NM	437(24.0%)	86.2	82.9 [79.7, 82.3]	74.8	69.1 [66.4, 71.8]
LMM	81 (4.5%)	96.3	95.2 [69.5, 79.1]	88.9	86.2 [81.4, 91.0]
ALM	150 (8.2%)	81.3	74.3 [92.5, 97.9]	65.3	56.2 [51.2, 61.7]
Other	158 (8.7%)	95.6	93.2 [87.9, 98.5]	89.2	83.8 [76.2, 91.4]
**SLNB status**			P<0.001		P<0.001
negative	1697 (88.9%)	92.0	90.3 [88.5, 92.1]	83.3	80.6 [78.2, 83.0]
positive	212 (11.1%)	74.5	70.9 [63.3, 78.5]	54.2	46.0 [38.0, 54.1]

SD = Standard Deviation; IQR = Inter-Quartile Range; SSM = superficial spreading melanoma, NM = nodular melanoma, LMM = lentigious malignant melanoma, ALM = Acral lentigious melanoma; AJCC = American Joint Committee of Cancer.

### Prognostic factors in SLNB staged patients

**Figure 1:**
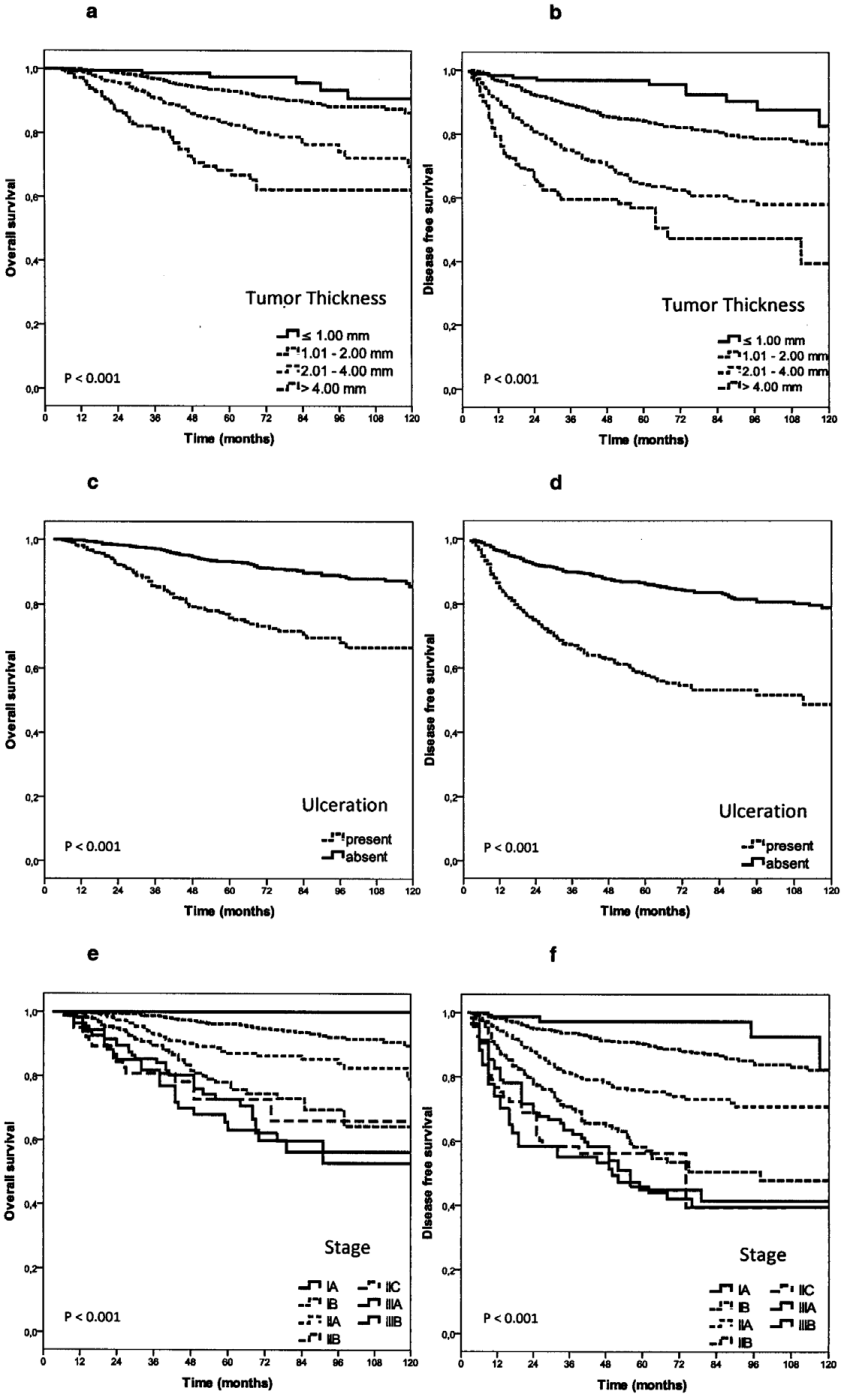
Survival in sentinel node staged patients. a) Overall survival according to primary tumor thickness (p<0.001). b) Disease free survival according to primary tumor thickness (p<0.001). c) Overall survival in patients with and without ulcerated primary tumors (p<0.001). d) Disease-free survival in patients with and without ulcerated primary tumors (p<0.001). e) Overall survival according to AJCC stage of primary tumor (p<0.001). f) Disease-free survival according to AJCC stage of primary tumor (p<0.001).

Kaplan-Meier analysis of OS identified age (p = 0.035), tumor thickness (p<0.001), body site (p<0.001), ulceration (p<0.001), Clark level of invasion (p<0.001), histological subtype (p<0.001) and SLNB status (p<0.001) as significant prognostic factors (see Table 1 and Figure 1a–f). Similar results were found for DFS for age (p = 0.001), tumor thickness (p<0.001), body site (p<0.001), ulceration (p<0.001), Clark level of invasion (p<0.001), histological subtype (p<0.001) and SLNB status (p<0.001) (see Table 1).

**Figure 2:**
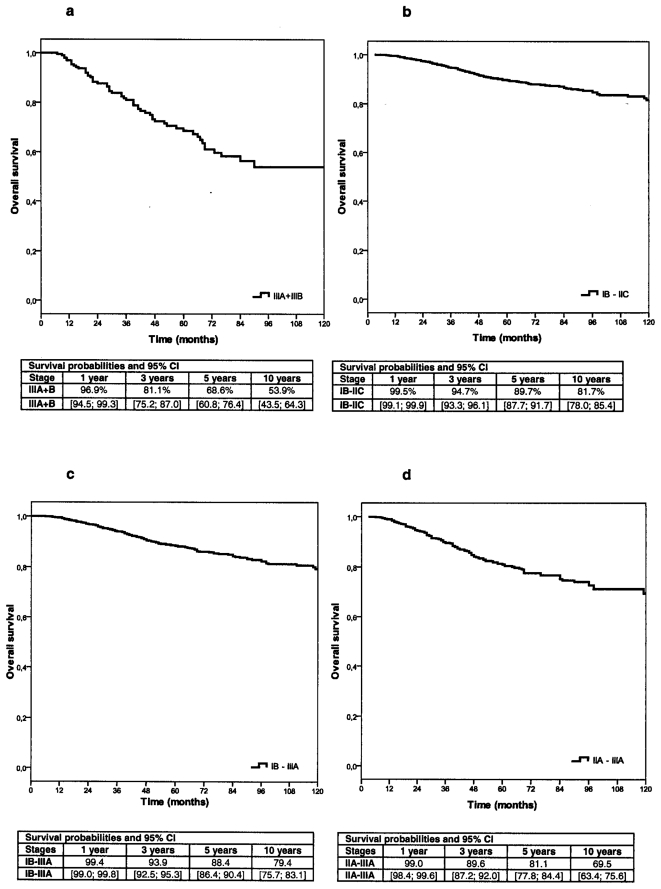
Overall survival in different stages of primary tumors according to AJCC 2009. a) Overall survival in stage IIIA CM patients. b) Overall survival in stage IB- IIC CM patients. c) Overall survival in stage IB- IIIA CM patients. d) Overall survival in stage IIA-IIIA CM patients.

The 5-year melanoma-specific survival probabilities were 90.3% (95%CI: 88.5; 92.1) for node-negative patients compared to 70.9% (95%CI: 63.3, 78.5; p<0.001) for node-positive patients (see Table 1, Figure 2a–d). The 5-year DFS for SLNB negative patients was 80.6% (95%CI: 78.2, 83.0), vs 46.0% (95% CI: 38.0, 54.1) for SLNB positive patients.

Analyzing stages according to AJCC 2009, no significant differences could be found for OS in stages IB-IIC compared to IB-IIIA, the 5-year OS was 89.7% and. 88.4%, respectively, Figures 2b–c. Stage IIC patients showed a similar 5-year OS as stage IIIA patients (72.6%), the DFS was 50.7% and 45.9%, respectively, see Table 1. The results of the current study are listed and compared to those of Balch et al. [15] in Table 2.

**Table 2:** Five and 10-year melanoma-specific survival in sentinel node-staged cutaneous melanoma patients (n = 1909). compared to in 27,000 stage I/II and 2,587 stage IIIA/B patients reported by Balch et al. [15], [18].

Stage	5-year OS		10-Years OS	
	Balch	Present Study	Balch	Present study
IB	n.g*	96%	n.g.	89.5%
T1b			85%	
T2			80%	
IIA	79–82%	87.0%	n.g.	79.2%
IIB	68–71%	78.1%	n.g.	64.3%
IIC	53%	72.6%	n.g.	66.0%
Micrometastases	67%	70.9%	n.g.	53.9%
IIIA	78%	72.6%	n.g.	56.4%
IIIB	59%	65.6%	n.g.	52.8%

*: n.g.: not given.

Multivariate Cox proportional hazard analyses identified tumor thickness, ulceration, body site, histological subtype and SLNB status as independent significant prognostic factors for melanoma-specific and disease-free survival, Table 3. Tumors with a thickness of >4 mm had an increased relative risk to die of CM (5.2, 95%CI: 2.1, 12.7) compared to CM of ≤1.0 mm thickness (p<0.001, Table 3). Patients with positive SLNB status were 2.3 (95%CI: 1.6, 3.1) times more likely to die from melanoma compared to patients with negative SLNB (p<0.001). Age, gender and Clark's level of invasion failed to be independent significant prognostic factors for overall and disease-free survival.

**Table 3:** Prognostic factors of overall (OS) and disease-free (DFS) survival in sentinel node-staged CM patients (n = 1909). Results of multivariate Cox proportional hazard analysis.

	OS#			DFS##		
Prognostic factor	RR	95% CI	p value	RR	95% CI	p value
**Tumor thickness**			<0.001			<0.001
≤1.0 mm	1			1		
1.01–2.0 mm	1.6	0.69, 3.8		2.1	1.1, 3.8	
2.01–4.0 mm	3.1	1.3, 7.2		3.8	2.0, 7.2	
>4.0 mm	5.2	2.1, 12.7		5.7	3.0, 11.1	
**Ulceration** *			<0.001			<0.001
Absent	1			1		
Present	2.1	1.5, 3.0		2.2	1.7, 2.9	
**Body site**			<0.001			<0.001
Upper limb	1			1		
Head and Neck	3.8	1.7, 8.3		3.1	1.9, 5.0	
Lower limb	2.5	1.3, 4.9		2.4	1.6, 3.6	
Trunk	4.9	2.5, 9.6		2.9	1.9, 4.3	
**SLNB status**			<0.001			<0.001
Negative	1			1		
Positive	2.3	1.6, 3.1		2.3	1.8, 3.0	
Histological subtype**			= 0.034			= 0.014
SSM	1			1		
NM	1.1	0.73, 1.5		0.82	0.63, 1.1	
LMM	0.72	0.20, 2.5		0.56	0.26, 1.2	
ALM	2.2	1.3, 3.7		1.3	0.91, 1.9	
Other	0.64	0.29, 1.4		0.54	0.32, 0.91	
**Gender**			= 0.422			= 0.639
**Age**			= 0.166			= 0.133
**Clark's level**			= 0.823			= 0.747

RR = hazard ratio; 95% CI = 95% confidence interval.

*Adjusted for 304 missing values; Adjusted for 90 missing values;

# Model for melanoma-specific survival was adjusted for the confounding effects of age and gender;

## Model for disease-free survival was adjusted for the confounding effect of age.

## Discussion

In the last two decades, sentinel lymph node biopsy has become a standard procedure for nodal staging in patients with primary CM and clinically uninvolved lymph nodes. If SLNB was not part of the management of a CM patient, this patient might not be considered eligible for clinical trials [1]. This exclusion would not only slow the development of more effective therapies but could also disadvantage patients by preventing them from receiving adequate therapy [1].

The current study was performed in order to evaluate survival probabilities and prognostic factors in 1909 SLNB staged CM patients. Only few cohorts of patients with primary CM and complete nodal staging with SLNB have been published so far [2], [12], [13], [19]. In most published cohorts analyzing survival, nodal staging was either incomplete or not comparable to the present results [3], [16], [19]. We found more favorable survival probabilities compared to previously published cohorts which had incomplete nodal staging [18]. In addition, we calculated survival probabilities for groups of patients with different stages who were at elevated risk for recurrences and who might be eligible for adjuvant treatments. These stage-specific survival probabilities may be useful for sample size calculations for future melanoma trials.

In the 2009 AJCC staging classification Balch and co-workers investigated 27000 CM patients in stage I/II and 2313 in stage III with complete follow-up data in the AJCC melanoma staging database [15], [18]. For the 27000 stage I/II patients with primary CM, tumor thickness, mitotic rate and ulceration were the most dominant prognostic factors.

Comparing the results of Balch et al. [18] to those of the present study, our patients showed an advanced 5-/10 year survival-probability, see Table 2. These discrepancies and improved survival for stage IB-IIC in our analysis may be caused by the fact that the data of 27000 patients in stages I/II published by Balch et al [18], may also include patients who had not undergone SLNB and were not truly negative for micro-metastases [18]. In particular, in stages IIB-IIC the discrepancies between our results and Balch's are greatest (Table 2). However, in these stages the 27000 patients were at the highest risk of unrecognized micro-metastases [18].

Several studies on prognostic factors in primary melanoma patients with negative SLNB have been published in the last years [3], [18], [19] confirming the impact of tumor thickness and ulceration. Yee et al reported a similar 5-year OS of 90% for all SLNB negative patients, ranging from 78% to 94% for patients with or without ulceration which is nearly identical to our results (Table 1a,b) [3]. In the present study SLNB containing isolated positive tumor cells or micrometastases of ≤0.1 mm were not judged as positive. This is in accordance to a recent publication from Eggermont's group [20]. In this study of 1,080 patients from van der Ploeg et al the 5 years OS of 91% in patients with micrometastases <0.1 mm in diameter was shown to be similar to those of SLNB negative patients. These results are in accordance to the five-years OS (90.6%) in SLNB negative patients in the current analysis [20]. Patients with micrometastases from 0.1 to 1.0 mm showed a similar 5-years OS of 74% [20] than stage IIIA patients in our study (72.6%).

Ulceration remains the second important prognostic factor associated with unfavorable survival [7], [15], [16], [18]. Survival rates of patients with an ulcerated CM were previously found similar to those of patients with a non-ulcerated CM of the next higher tumor thickness category [15], [18]. This is in concordance to our results, where ulceration proofed to be an independent significant prognostic factor in primary CM and in the total collective of SLNB staged patients (Tables 1, 3 and Figure 1c,d).

In our collective of positive and negative SLNB staged patients, SLNB status, tumor thickness, ulceration, histological subtype and body site were identified as independent significant prognostic factors for DFS and OS during multivariate analyses. In order to define subgroups for clinical trials, histological subtype and body site may provide additional prognostic information. The SLN status was shown to be a highly significant prognostic factor with a 5-year OS of about 90.3% in negative SLNB and 70.9% in positive SLNB. The impact of the SLN status, tumor thickness and ulceration on DFS and OS has been confirmed by various publications [3], [4], [12], [13]. In addition, histological subtype was reported to be a further significant independent prognostic factor [21], [22] for OS and DFS. Studies in SLNB negative patients did not show this effect [3]. In patients with stage III disease body site was shown to be a further prognostic factor [15].

One limitation of the present study is the missing information for mitotic rate. Until January 2010, the mitotic rate of primary melanoma was not determined in Germany. However we do not expect that this limitation would change our results markedly. A positive mitotic rate upstages patients with tumor thickness less than 1.0 mm which would then be classified as stage IB instead of IA. In our collective the number of patients with tumor thickness less than 1 mm was small (5.3%), and hence a marked change of the OS and DFS is unlikely.

### Conclusion

The current study was performed to evaluate the survival probabilities and prognostic factors of 1909 SLNB staged CM patients. Five year survival rates for different subgroups eligible for adjuvant trials were found to be quite favourable (five years Overall Survival rates of 89.7% in stages IB – IIC, 88.4% in stages IB – IIIA, 81.1% in stages IIA – IIIA, 68.8% in stages IIIA/B). The prognosis for primary CM patients without micro-metastases in SLNB seems to be more favorable than previously reported. It is suggested that this is mainly due to the exclusion of sentinel-node positive patients from stages I–II. It is necessary to adapt sample size calculations for survival probabilities classified by the presently valid AJCC staging system.

## Supporting Information

Supporting Information S1 **Informed consent.**
(PDF)

Supporting Information S2 **Statement of the local Ethic Committee.**
(PDF)
