# Supplementary material for: Prognosis of Sentinel Node Staged Patients with Primary Cutaneous Melanoma
Source: PLoS One. 2012 Jan 19;7(1):e29791. doi: 10.1371/journal.pone.0029791 (PMC3261856; doi:10.1371/journal.pone.0029791)
Supplement: Supporting Information S1 — Informed consent. (PDF) [file pone.0029791.s001.pdf]

EBERHARD KARLS
UNIVERSITÄT
TÜBINGEN

Eberhard-Karls-Universität UKT Universitätsklinikum Tübingen

Medizinische Fakultät

Ethik-Kommission

Prof. Dr. med. D. Luft
Vorsitzender

Telefon: +49 7071 29-77661
Telefax: +49 7071 29-5965
E-Mail:
ethik.kommission@med.uni-tuebingen.de

Ethik-Kommission an der Medizinischen Fakultät der Eberhard-Karls-Universität und am Universitätsklinikum Tübingen, Gartenstraße 47, 72074 Tübingen

Frau
PD Dr. med. Ulrike Leiter-Stöppke
Abteilung Dermatologie
Universitäts-Hautklinik
Liebermeisterstraße 25
72076 Tübingen

nachrichtlich:
Herrn Prof. Dr. med. Martin Röcken
Herrn Prof. Dr. med. C. Garbe

**244/2011A**	2. Mai 2011	04. Mai 2011
unsere Projekt-Nummer	eingegangen am	Datum

Prognosis of sentinel node staged patients with primary cutaneous melanoma.
Abstract
erläuterndes Begleitschreiben vom 20.04.2011

Sehr geehrte Frau Kollegin,

Ihre Anfrage bezüglich der retrospektiven Auswertung bereits vorhandener Daten von Patienten, die im Zweitraum zwischen 1996 und 2009 unter der oben genannten Diagnose in der Universitäts-Hautklinik untersucht und behandelt wurden, hat der Ethik-Kommission zur Beratung vorgelegen.

Die retrospektive, anonymisierte Auswertung individueller, in der Diagnostik und Therapie entstandener Daten eigener Patienten bedarf keiner Beratung durch die Ethik-Kommission gemäß der Berufsordnung für Ärzte und keiner informierten Einverständniserklärung der früher untersuchten Patienten.

Gegen eine anonymisierte Zusammenstellung, Auswertung und Publikation der Daten bestehen seitens der Ethik-Kommission keine Bedenken.

Mit freundlichen Grüßen

Prof. Dr. med. Dieter Luft
Vorsitzender der Ethik-Kommission

Seite 2: Allgemeine Hinweise zum Votum der Ethik-Kommission

Universitätsklinikum Tübingen
Anstalt des öffentlichen Rechts
Sitz Tübingen
Geissweg 3 · 72076 Tübingen
Telefon +49 7071 29-0
www.medizin.uni-tuebingen.de
Steuer-Nr. 86156/09402
USt-ID: DE 146 889 674

Aufsichtsrat
Klaus Tappeser
(Vorsitzender)

Vorstand
Prof. Dr. Michael Bamberg (Vorsitzender)
Gabriele Sonntag (Stellv. Vorsitzende)
Prof. Dr. Karl Ulrich Bartz-Schmidt
Prof. Dr. Ingo B. Autenrieth
Jana Luntz

Banken
Baden-Württembergische Bank Stuttgart
(BLZ 600 501 01) Konto-Nr. 7477 5037 93
IBAN: DE41 6005 0101 7477 5037 93
SWIFT-Nr.: SOLADEST
Kreissparkasse Tübingen
(BLZ 641 500 20) Konto-Nr. 14 144
IBAN: DE79 6415 0020 0000 0141 44
SWIFT-Nr.: SOLADES1TUB
