# Supplementary material for: Prognosis of Sentinel Node Staged Patients with Primary Cutaneous Melanoma
Source: PLoS One. 2012 Jan 19;7(1):e29791. doi: 10.1371/journal.pone.0029791 (PMC3261856; doi:10.1371/journal.pone.0029791)
Supplement: Supporting Information S2 — Statement of the local Ethic Committee. (PDF) [file pone.0029791.s002.pdf]

Zentralregister Malignes Melanom
der Deutschen Dermatologischen Gesellschaft

Koordinationsstelle: Eberhard-Karls-Universität, Universitäts-Hautklinik
Liebermeisterstr. 25, 72076 Tübingen
Meldestelle: Freie Universität Berlin, Universitäts-Hautklinik
Fabeckstr. 60 – 62, 14195 Berlin

Kontakte: Prof. Dr. C. Garbe, Dr. U. Leiter, M. Kreißig
Tel.: **07071/29-** 87110, -80833 ● Fax -5187
email: michael.kreissig@med.uni-tuebingen.de

Patienteninformation zur Meldung von Daten an das Zentralregister Malignes Melanom

Verehrter Patient, verehrte Patientin!
In den letzten Jahren wurden von allen Universitäts-Hautkliniken und vielen Hautärzten in der Bundesrepublik vermehrt Hautveränderungen und Tumoren beobachtet, die dunkel pigmentiert sind und aus Leberflecken entstehen oder diesen ähneln.

Bisher ist nur wenig über die Ursachen der zunehmenden Häufigkeit dieser Hautveränderungen bekannt. Eine optimale Vorsorge, wie auch ihre Behandlung erfordern jedoch eine genaue Erfassung ihres Vorkommens und eine wissenschaftliche Auswertung.

Um die Entwicklung solcher Hautveränderungen besser verstehen zu lernen, vorbeugende Maßnahmen ergreifen zu können und verbesserte Therapien zu entwickeln, wurde von der Deutschen Dermatologischen Gesellschaft ein Spezialregister eingerichtet. Dieses Register ist im Universitätsklinikum der Eberhard-Karls-Universität in Tübingen angesiedelt. Es erfasst genaue Angaben über die Art der Krankheit, Größe und Ausdehnung der Flecken und Tumoren, die Wohngegend der Kranken, sowie Angaben zur durchgeführten Therapie. Um Folgemeldungen über die Entwicklung der Krankheit zuordnen zu können und Doppelmeldungen zu vermeiden, werden die zu registrierenden Daten mit Namen und Anschrift der Patienten erfasst. Ihre persönlichen Daten werden streng vertraulich behandelt und **nicht** veröffentlicht. Alle Informationen, die mit Ihnen in Zusammenhang gebracht werden können, werden geheim gehalten und nur mit Ihrer Erlaubnis weitergegeben. Sollten Sie dies nicht wünschen kann auch eine anonyme Registrierung beim Zentralregister Malignes Melanom erfolgen. Falls es Sie interessiert, können Sie jederzeit darüber Auskunft erhalten, welche Ihrer Daten erfasst sind. Die Deutsche Dermatologische Gesellschaft ist der Ansicht, dass jeder Patient über ein derartiges wissenschaftliches Programm umfassend aufgeklärt werden soll. Die Einholung seines Einverständnisses trägt zur Erhaltung und Verbesserung des Vertrauensverhältnisses zwischen Arzt und Patienten bei. Wir bitten Sie um Ihre Mitarbeit und Ihr Einverständnis.

Einverständniserklärung

Ich wurde darüber aufgeklärt, dass Daten zu meiner Hauterkrankung an das Spezialregister der Deutschen Dermatologischen Gesellschaft weiter geleitet werden. Dieses Register erfasst aus Gründen der Zuordnung auch Name und Anschrift mit, die unter ärztlicher Aufsicht verbleiben. In Folgemeldungen wird die weitere Entwicklung der Krankheit aufgezeichnet werden. Die beteiligten Personen werden dabei die Bestimmungen des Datenschutzgesetzes und die ärztliche Schweigepflicht einhalten. Die Daten werden ausschließlich zu wissenschaftlichen Zwecken mit dem Ziel besserer Vorsorge und besserer Behandlung der Hautkrankheiten verwendet.
Ich erkläre zu diesem Vorgehen mein Einverständnis.

……………….. …………………. …………………………..

Ort Datum Unterschrift
